# Noise rejection through an improved quantum illumination protocol

**DOI:** 10.1038/s41598-021-01122-8

**Published:** 2021-11-08

**Authors:** T. Gregory, P.-A. Moreau, S. Mekhail, O. Wolley, M. J. Padgett

**Affiliations:** 1 School of Physics and Astronomy, University of Glasgow, Glasgow, G12 8QQ UK; 2 Department of Physics, National Cheng Kung University, Tainan, 70101 Taiwan; 3 Center for Quantum Frontiers of Research and Technology, NCKU, Tainan, 70101 Taiwan

**Keywords:** Quantum optics, Nonlinear optics

## Abstract

Quantum illumination protocols can be implemented to improve imaging performance in the low photon flux regime even in the presence of both background light and sensor noise. However, the extent to which this noise can be rejected is limited by the rate of accidental correlations resulting from the detection of photon or noise events that are not quantum-correlated. Here we present an improved protocol that rejects up to $\gtrsim 99.9\%$ of background light and sensor noise in the low photon flux regime, improving upon our previous results by an order of magnitude. This improvement, which requires no information regarding the scene or noise statistics, will enable extremely low light quantum imaging techniques to be applied in environments previously thought difficult and be an important addition to the development of covert imaging, quantum microscopes, and quantum LIDAR.

## Introduction

Spatial correlations arising from the quantum entanglement of photon-pairs can be harnessed to obtain a variety of quantum enhancements in imaging^1–4^. These enhancements are usually achieved through implementing an algorithm to preferentially select the detected photon-pair events and reject all single events whether these single events be background photons or sensor noise. However, any coincidence detection algorithm may also select event-pairs that do not correspond to genuine correlations between an entangled photon-pair, rather between randomly correlated single events. The collection of these random correlations results in the degradation of the final quantum-enhanced image. These random correlations can be increased further by a third party projecting a spoof image or scene to either interfere with, or to mislead, the imaging system. In this situation some proportion of this spoof illumination will remain in the quantum enhanced image. Solving such issues is of importance in further improving the performance of quantum imaging techniques to allow quantum imaging to be performed at low illumination levels under potentially hostile or adverse conditions. Imaging at extremely low light levels in the presence of high levels noise and loss has applications in quantum lidar and covert imaging applications. These technologies will see deployment in scenarios in which it is necessary that it is difficult for the target to determine that it is being imaged thereby requiring a low light level for the active illumination. Furthermore, these scenarios will present a hostile imaging environment in which active spoofing is present in addition to environmental noise and losses. Further to these technologies, the principle of removing the random coincidences between random events may inform the development of applications in secure information transmission and non-invasive biological imaging.

In this work we implement a quantum illumination protocol to select the events that result from the detection of entangled photon-pairs, while preferentially rejecting accidentally correlated events caused by both background light and sensor noise. Quantum illumination protocols were initially proposed by Lloyd^5^, and Tan et al.^6^ to confirm the presence, or absence, of a target object within a noisy and lossy environment^7–9^. Subsequently, these quantum illumination protocols have been experimentally demonstrated in the optical regime using the spatial correlations^10–12^ and in the near-infrared regime using the spectro-temporal correlations^13^ between photon-pairs. Quantum illumination protocols have also been proposed in the microwave regime for quantum radar applications^14–16^. These quantum illumination protocols are resilient to both noise and losses and the advantage is increased in high noise and high loss environments as previously theorised^17^. This resilience is unlike conventional quantum correlation imaging techniques which, while capable of surpassing classical limits of imaging^1, 3, 4, 18–22^, are susceptible to the effects of decoherence caused by the introduction of noise and losses into the system. This susceptibility renders many conventional quantum imaging techniques of limited real-world benefit as they remain restricted to low-noise and low-loss conditions, and therefore only to the sensing of objects presenting relatively low absorption. Quantum illumination protocols, on the other hand, allow a quantum enhancement to be realised even under such adverse conditions such as the high noise environment that we explore here. Our purpose here is to further improve such protocols to enable real-world quantum enhanced imaging systems.

In our previous work^12, 23^ we implemented a quantum illumination protocol and applied it to full-field imaging using a detector array positioned in the focal plane. In that experimental implementation the spatially correlated photon-pairs that comprise the probe and reference beams were produced using the spontaneous parametric downconversion (SPDC) process within a BBO crystal cut for type-II phase-matching. The entangled photon-pairs that comprise the probe and reference beams are spatially separated in the far-field of the downconversion crystal due to the crystal birefringence. The photons in the probe arm illuminate the object while those in the reference arm have a free optical path through this far-field plane in which the object is located. The spatially correlated photon-pairs that comprise the probe and reference beams were relayed onto neighbouring regions of an Electron Multiplying CCD (EMCCD) array detector placed in the far-field plane of this crystal and the image plane of the object. The illumination level and the EMCCD detector were set such that each frame was sparsely populated after thresholding was performed. This allows single photon detection events to be recorded and the spatial correlations between detected single photon events to be used to realise the enhancement. Previously EMCCD array detectors have also been used to measure the spatial correlations between entangled photon-pairs in both the image plane of the downconversion crystal and also the far-field and also to demonstrate the presence of entanglement^24–26^. For each acquired frame a pixel-by-pixel AND-operation between the two regions of the array detector upon which the probe and reference beams are recorded is performed to identify the spatially correlated photon-pair events. This operation also serves to reject events resulting from uncorrelated background light and sensor noise. The quantum illumination AND-image is calculated from the sum of all these AND-events, acquired over a large number of frames, while the classically acquired image comprises the sum of all the events. In our earlier work^12^ a thermally illuminated mask was used to overlay a spoof image on top of a true image of a mask illuminated by SPDC illumination. We showed a preferential rejection of the spoof image and improved our ability to distinguish the true image from the spoof image by up to a factor of 4. However, as the thermal illumination increased, the number of false correlations kept in the quantum illumination AND-image also increased meaning that the classically illuminated component of the image could not be fully suppressed.

To assess a new imaging protocol we set a baseline against which it is to be compared. The quantum advantage that is reported in this work and also in^12^ is relative to the corresponding classical measurement that is obtained by photon-counting and is not obtained by performing a phase-sensitive measurement in either a heterodyne or homodyne detection scheme. Whilst other classical imaging schemes against which the quantum advantage could be measured, such as those using classical correlations^10^ or phase-sensitive measurements, could outperform the imaging scheme presented here, they would require operating under an increased photon flux and therefore within a differing imaging regime. In the context of a photon-flux limited quantum lidar or covert imaging scheme an assessment of the advantage under identical illumination conditions is required. Therefore, in order to assess the quantum advantage, the classically acquired image and the quantum illumination AND-image are obtained under identical experimental conditions and within the same acquisition thereby providing a direct comparison between equivalent quantum and classical imaging schemes. The requirements of a phase-sensitive implementation^6, 7^ would be that a much more complicated optical setup is used for the acquisition of the classical data.

In this present work we improve upon our previously reported result using information contained within both the quantum illumination AND-image and the classically acquired image alongside the information contained within the reference beam to improve the final image beyond that of the quantum illumination AND-image alone. The combination of the quantum and classical information allows the removal of accidental AND-events that are present in the AND-image due to the presence of noise and losses in the imaging system. The resulting image demonstrates an advantage in the distinguishability ratio of up to 27 relative to the classically acquired image by largely removing the structured illumination spoof image regardless of its intensity. The advantage of using this combination of classical and quantum information is that all the data required to perform the background subtraction is obtained within the same acquisition under the same experimental conditions. There is no requirement to calibrate the system or to characterise the background light independently with respect to either its structure or the underlying statistics and no prior information is required. Using a combination of the quantum and the classical information to perform the subtraction of accidental correlations allows a better image of the true scene to be revealed, a feat that could not be achieved by using an arbitrary background subtraction which would result in a final image that misrepresents the true scene. The resulting improvement in the final image may allow quantum imaging to be performed in real-world environments where sensor noise, background illumination, and spoof signals all require suppression. Other schemes that utilise the combination of quantum and classical information have been previously implemented and demonstrated to enhance imaging over classical imaging schemes^27^. In the context of quantum experiments in the temporal domain the subtraction of accidental coincident events is a component of established characterisation methods for sources of entangled photon-pairs in the calculation of the coincidence-to-accidental ratio^28, 29^.

## Methods

### Imaging system

**Figure 1:**
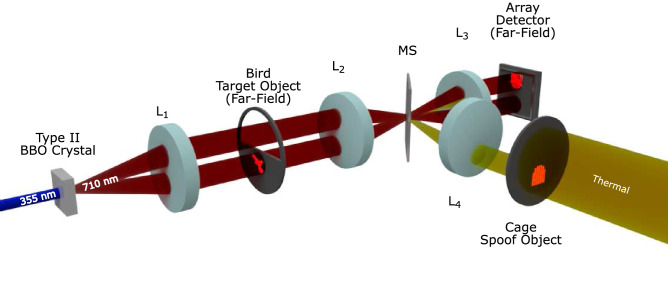
The quantum illumination experimental setup. A UV laser pumps a BBO crystal cut for type-II phase-matching to generate entangled photon-pairs via SPDC. The probe beam interacts with the bird target object placed in the far-field of the downconversion crystal while the reference beam has a free optical path. The lens system $L_{1} = 75 \text { mm, } L_{2} = 400\text { mm, and } L_{3} = 50\text { mm }$ forms the arm in which the quantum light propagates and serves to demagnify the far-field plane in which the bird resides by a factor of eight onto the EMCCD camera array detector. A thermally illuminated cage spoof object is also projected onto the camera over the region on which the probe beam is detected using lenses $L_{4} = 300 \text { mm and } L_{3} = 50\text { mm }$ by reflection from the microscope slide slip cover (MS).

The experimental configuration identical to that which we reported in^12^ is summarised here and shown in Fig. 1. A laser beam of wavelength $355 \text { nm}$ is used to generate SPDC photon-pairs centred on the degenerate wavelength of $710 \text { nm}$ by pumping a $3 \text { mm}$ long $\beta$-barium borate (BBO) non-linear crystal cut for degenerate downconversion and type-II phase-matching. Due to the photon source being cut for type-II phase-matching the photon-pairs are emitted with orthogonal polarisation and as a result the probe and reference beams are separated in the far-field plane of the downconversion source due to the birefringence of the downconversion crystal. The two beams generated by these photon-pairs are labelled as the probe beam, which interacts with the object in the far-field of the downconversion source and contaminated with background light, and the reference beam, which passes through the same optical system but which bypasses the object and does not experience this background light. To reduce autofluorescence of optical components the remainder of the pump beam is removed after transmission through the downconversion source by a pair of dichroic mirrors. The downconverted photon-pairs generated at degenerate wavelength $710 \text { nm}$ are selected by interference filters centred at $710 \text { nm}$ with a bandpass of width $10 \text { nm}$. The far-field plane of the crystal is then imaged onto the EMCCD camera after demagnification by a factor of eight. Further demagnification in order to match the size of the correlation peak to the detector pixel size would have resulted in images that are overly pixelated. The image of the thermally illuminated cage is overlaid on the probe beam by reflection from a microscope slide slip cover (MS) onto the detector. For our implementation the thermally illuminated spoof image is deliberately introduced separately from the probe beam. Were the background light to be introduced prior to the object being probed the detected background light would be modulated by the object. In our experimental configuration the reference and the probe beam pass through the same optical system and are not physically separated into two different arms. Operating with the object and the detector in the far-field of the downconversion crystal is a configuration that allows the spoof image to overlay only one of the beams, that is the probe beam. However, in the far-field there is a broadening of the correlation peak due to the finite bandwidth of the source thereby limiting the achievable resolution^30, 31^. Operating in the image plane of the downconversion crystal the photon-pairs could be separated by polarisation into two separate imaging arms, one for the probe, one for the reference and for the spoof image to be introduced into the image plane of the downconversion crystal but only for one of the beams. Further to an increase in resolution, operating in the image plane of the downconversion crystal the photons of differing wavelengths overlap within the SPDC beam rather than forming rings which could enable multispectral covert imaging to be performed.

In the case of our implementation the size of the correlation peak (FWHM $3.51 \pm 0.12$ pixels) extends beyond a single pixel and therefore many correlated photon-pair events are not collected. This effectively introduces additional losses into the system but is somewhat counteracted in this work by our performing the AND-operation over a $3 \times 3$ grid to collect an increased proportion of photon-pair events but at the expense of collecting increased accidentally coincident events. This operation is performed in a stepped manner so as to avoid double counting of detector events.

The camera used here is an Andor iXon ULTRA 888 DU-888U3-CS0-#BV; of pixel size $13\times 13 \mu \text {m}^2$, $100\%$ fill-factor EMCCD camera. The camera was cooled to $-90^{\circ }\text {C}$ using peltier and water cooling. Optimal acquisition parameters for the camera were set as follows: vertical speed $1.33 \text { }\mu \text {s}$; voltage clock amplitude $+0 \text { V}$, horizontal speed $10 \text { MHz}$; EM gain 1000; pre-amplifier gain set to 1; $128 \times 128$ pixel acquisition region; exposure time of $0.0149500 \text { s}$. For the given acquisition parameters the shortest exposure time allowable was used to maximise the data rate. The illumination regime in which the data was obtained was that in which the number of thresholded SPDC photon detection events was similar to the number of thresholded events resulting from camera noise. This regime was shown by Tasca et al. (2013)^32^ as the regime in which the visibility of the correlation signal is maximised. Under our operating conditions the dark event rate is of order $\sim 0.0016$ events per pixel per frame and so SPDC illumination is set to give a total event rate of $\sim 0.0032$ events per pixel per frame. A threshold was applied to each of the frames acquired by the EMCCD camera in order to minimise the number of camera readout noise events which present themselves at low values of the analogue-digital converter counts while maximising the quantum efficiency. For all pixels this threshold was set at $T \approx \mu + 3.5 \sigma$ where $\mu$ and $\sigma$ are respectively the mean and standard deviation of the detector readout signal for dark frames.

### Image analysis

To preferentially select spatially entangled photon-pair events over uncorrelated noise events, a pixel-by-pixel AND-operation is performed between the two regions of the sensor that detect the reference and the probe beams within each frame. In the far-field, the detected photon-pair events occur in positions rotated through an angle of $\pi$ about the pump beam axis. As a result, of the two regions of the camera on which the reference and probe beam are detected, one is rotated through $\pi$ prior to performing the AND-operation. The AND-operation is performed on each frame and the results are summed to construct the quantum illumination AND-image $I_{AND}$. For comparison, and subsequent processing, the classical images of the probe beam $I_{C}$ and the reference beam $I_{ref}$ are obtained by summing all events that are registered by the detector with analogue counts above our threshold.

The correlation peak between the probe and reference beams is of FWHM $3.51 \pm 0.12$ pixels and therefore extends beyond a single pixel which means that many of the photon-pair events are not perfectly anticorrelated in the pixels upon which they are detected. In order to collect an increased number of events that correspond to photon-pairs the AND-operation is also performed on the 8 pixels surrounding the central correlation position and as such the AND-operation is performed over a $3 \times 3$ grid. At each position the classical image of the probe beam $I_{C}$ and the reference beam $I_{ref}$ are obtained, and quantum illumination AND-image $I_{AND}$ calculated. At each step those events that have already been partnered are removed prior to the next step in order to avoid double counting and thereby prevent the introduction of additional noise events into the AND-images. Matching the area of the correlation peak relative to the area of the detector pixels would result in final images with a reduced shot-noise. Each of the quantum illumination AND-images $I_{AND}$ contains a contribution from the event-pairs that correspond to entangled photon-pairs $C_{q}$ and a contribution from accidental correlations $C_{u}$ as given in Eq. 1.

1 $$\begin{aligned} I_{AND} = C_{q}+C_{u} \end{aligned}$$

While the AND-operation is designed to preferentially select the correlated photon-pairs and reject uncorrelated events, accidental correlations between event pairs that do not correspond to entangled photon-pairs will also be selected as a result of spurious background light and noise. Accidentally correlated events are kept in $I_{AND}$ at a rate that equates to the product of the fill-factor of each pixel in the detected probe beam $\eta _{p}$ and the fill-factor of the corresponding pixel located in the reference beam $\eta _{ref}$. Consequently for each pixel in $I_{AND}$ the contribution from accidental correlations $C_{u}$ is as expressed in Eq. 2.

2 $$\begin{aligned} \begin{aligned} C_u&= \eta _{ref} \eta _{p} {\mathcal {N}}_f \\&= \eta _{ref} I_{C} \end{aligned} \end{aligned}$$

Where ${\mathcal {N}}_f$ is the number of acquired frames and $I_{c}=\eta _{p} {\mathcal {N}}_f$ is the number of photons accumulated in the corresponding pixel of the probe beam part of the sensor, that is the classical image. The value of the reference beam fill-factor is an already known quantity and therefore no additional acquisition or calibration step is required to obtain this information.

By performing the subtraction to each of the resulting AND-images as per Eq. 3 and then combining them into a single image, we show that an improvement in the distinguishability may be achieved through the suppression of these accidental correlations associated with the structured background image.

3 $$\begin{aligned} I_{Sub} = I_{AND} - \eta _{ref} I_{C} \end{aligned}$$

The removal of a structured background cannot be achieved through an arbitrary algorithmic background subtraction from the classical image without the AND-image because any attempt to arbitrarily subtract the unknown background will result in a final image that may misrepresent the actual scene. In the following we show that this procedure allows a better identification of the object and the ability to distinguish the real object from the background under the conditions in which we operate.

## Results

### Rejection of structured thermal illumination

Here we shall quantify the advantage in noise rejection and the distinguishability metric for the subtraction image generated by the above analysis relative to the classical image. As detailed in our previous work^12^ the noise rejection metric is the ratio of the average value of the quantum-illuminated target object regions, $\left\langle O\right\rangle$, and the average value of the classically illuminated spoof object regions $\left\langle S\right\rangle$. However, the ability to distinguish the quantum illuminated target object from the thermally illuminated spoof object is affected by the shot noise on the quantum illuminated object regions, $(\sigma _{O})$. The distinguishability metric, as defined in Eq. 4, considers this factor. In order to assess the distinguishability metric, *D*, for each of the images the average pixel value of the background of the images, that is the regions that comprise neither the bird nor the cage, is subtracted from all pixels in the image and the magnitude of the pixel values are used in calculating the aforementioned metrics.

4 $$\begin{aligned} D = \frac{\left\langle O\right\rangle }{\left\langle S\right\rangle + \sigma _{O} } \end{aligned}$$

**Figure 2:**
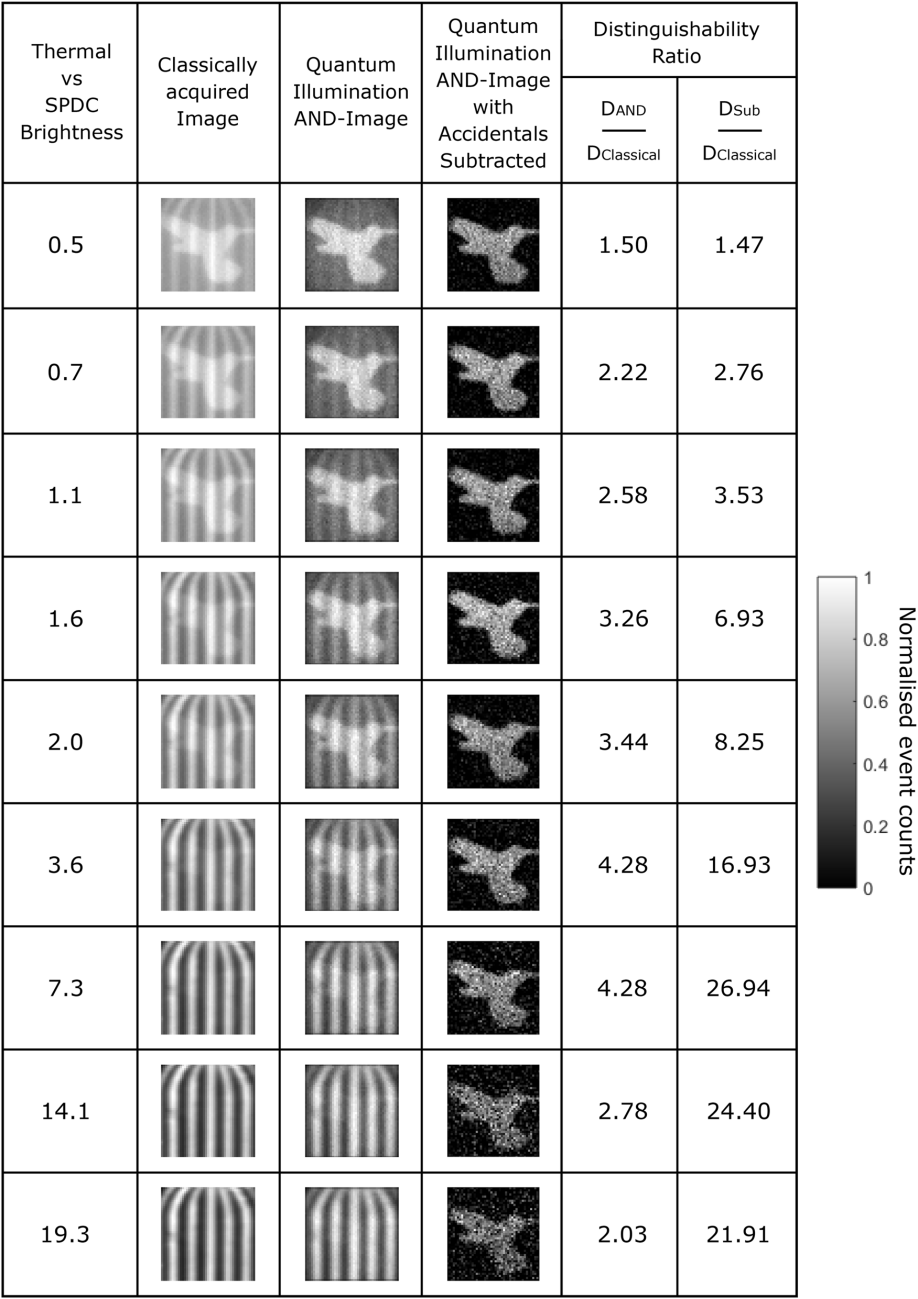
Images of the classically acquired image, the quantum illumination AND-image, and the quantum illumination AND-image with accidentals subtracted alongside their corresponding improvements in the distinguishability metric relative to that of the classically acquired image. Images constructed over 2.4 million frames. Data an extension of that presented in^12^.

In Fig. 2 we show a series of images in which the thermal illumination of the classically illuminated cage is increased from $\sim 0.5 \times$ to $\sim 20 \times$ that of the SPDC illumination of the bird. The classical image of the bird deteriorates as the illumination level of the classical cage is increased, as the spoof image is clearly visible across all levels of thermal illumination. The quantum illumination AND-image is always an improvement over the classical image as the relative intensity of the thermally illuminated cage compared to the quantum illuminated bird is reduced. This is due to the preferential rejection of sensor noise and the background light that comprises the cage by performing the AND-operation. However, in the quantum illumination AND-image some structure remains visible as a result of keeping accidental correlations between event-pairs which do not correspond to SPDC photon-pairs and the cage eventually becomes the dominant component of the quantum illumination AND-image at high background levels as represented in the bottom three rows. By performing the subtraction as described in the preceding section the thermally illuminated cage is largely rejected and the bird becomes visible across the full range of thermal illumination levels.

Across all levels of thermal illumination the ratio of the distinguishability of the quantum illumination AND-image with accidentals subtracted to the classical image $D_{Sub} / D_{Classical}$ is greater than that for the corresponding quantum illumination AND-image to the classical image $D_{AND} / D_{Classical}$ by up to a factor of $\sim 11$ when the thermally illuminated cage is $\sim 20 \times$ brighter than the bird illuminated by SPDC light. There is an increasing trend in the value of $D_{Sub} / D_{AND}$ as the level of thermal illumination increases across the range presented here as is expected in an application of the quantum illumination protocol and as we previously demonstrated^12^. The improvement in the distinguishability ratio for the quantum illumination AND-image with accidentals subtracted relative to the quantum illumination AND-image demonstrates the benefit of combining the quantum and the classical information as opposed to solely using either set of information alone.

In the case of the distinguishability ratios there is a decrease in these values as the level of thermal illumination relative to SPDC illumination continues to increase from $\sim 7 \times$ to $\sim 20 \times$. The decrease in these ratios is the result of the spillage of background light from the thermally illuminated cage into the regions of the image that are designated as belonging to the bird regions or the background regions of the image. For a bright thermal illumination source a pixel that is determined to have imaged part of the bird object and of value $n_{bird}$ then lies within the noise of the background regions $\sigma _{background}$ which affects the values of the noise rejection and distinguishability for the classical image. However, while the distinguishability ratios relative to the classical image decrease as the level of thermal illumination increases, the relative improvement in the distinguishability of the quantum illumination AND-image with accidentals subtracted compared to the quantum illumination AND-image increases from 6 to 11 across this range of thermal illumination thereby demonstrating the improvement in the quantum illumination AND-image with accidentals subtracted over the quantum illumination AND-image.

It can be seen in Fig. 2 that the quantum illumination AND-image and the quantum illumination AND-image with accidentals subtracted exhibit greater shot-noise than the classical image due to these images containing fewer events. In the case of the quantum illumination AND-image with accidentals subtracted this shot noise increases with increasing thermal illumination due to the increased number of events per pixel *n*, and correspondingly increased $\sqrt{n}$, within the classical image. These fluctuations are introduced into the quantum illumination AND-image with accidentals subtracted when the subtraction is performed resulting in an image with increased shot-noise. This introduction of additional shot-noise is inherent to the subtraction operation performed to combine the two images into the quantum illumination AND-image with accidentals subtracted and is particularly evident in the bottom two rows of Fig. 2. Despite this degradation, the use of the quantum illumination AND-image with accidentals subtracted still possesses an advantage over both the classical image and also the quantum illumination AND-image in terms of background noise rejection of the cage and also the distinguishability ratio at these higher thermal illumination to SPDC illumination ratios.

### Noise rejection in imaging a complex object

**Figure 3:**
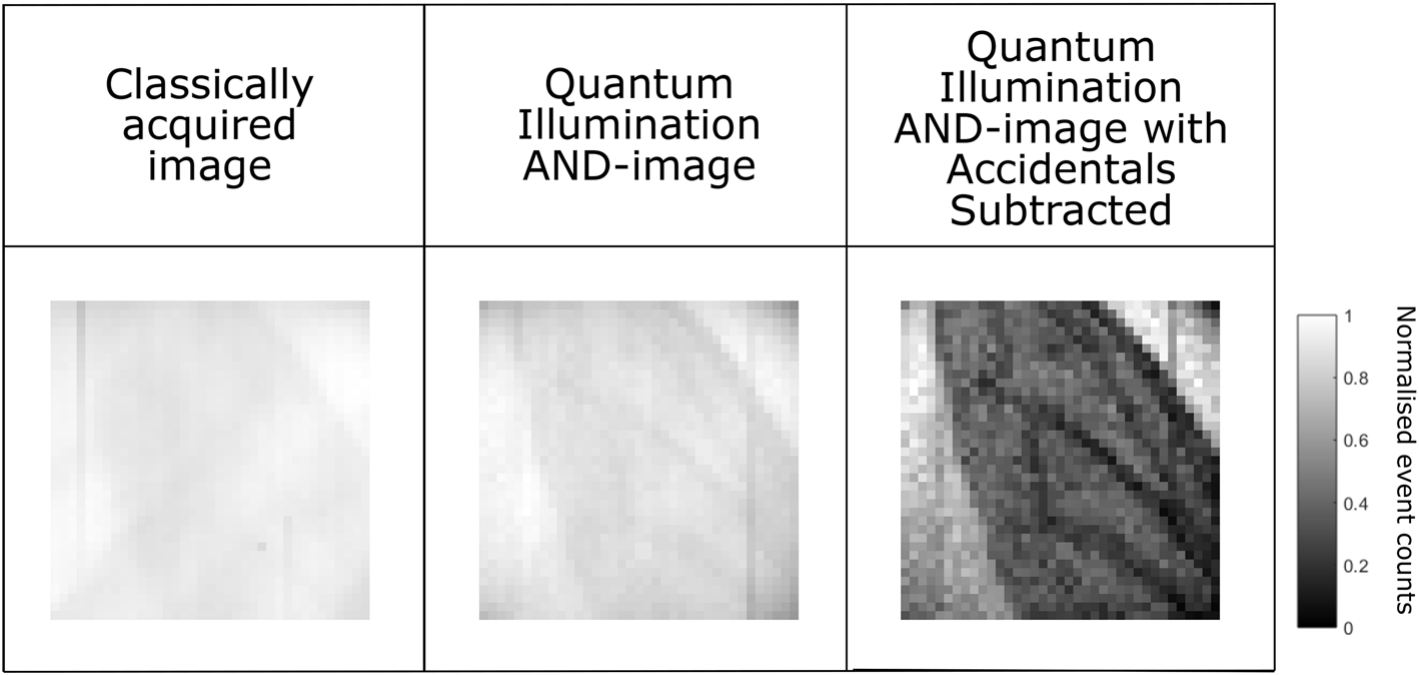
Wasp Wing. Images of the wasp wing. Classically acquired image (left), quantum illumination AND-image (centre), the quantum illumination AND-image with accidentals subtracted by combining the quantum and classical data (right). Images constructed over 87.7 million frames.

Here we demonstrate how our quantum illumination protocol combined with our new approach to rejecting the classical components of the image may be used to perform imaging of a real-world non-binary object presented in Fig. 3. In this case we image a wasp’s wing as the real object with an overlaid thermal beam that has passed through a dirty and scratched microscope slide. The thermal to SPDC illumination ratio for this data is $\sim 1.5$. As may be seen in the classical sum image the wing is barely visible and it is not possible to distinguish the structure of the wing. Application of the AND-operation to select the correlated event-pairs results in the quantum illumination AND-image in which the outline and the internal structure of the wing becomes partially visible but the features are of low contrast. After applying the subtraction of the accidental correlations to generate the quantum illumination AND-image with accidentals subtracted, as described above, the thermal light is largely removed and the internal structure of the wing becomes clearly visible with increased contrast.

## Conclusion

Here we demonstrated a significant improvement with regards to achievable background light and sensor noise rejection for a full-field quantum illumination protocol in a single photon regime, beyond that previously achieved, by using only information already available in the reference beam. The method of combining the quantum and classical information presented here may be limited in environments in which the background light signal is low or the heralding efficiency is high because then the shot-noise on the classical image will be close to that on the quantum illumination AND-image. In such cases performing a subtraction will lead to reduced image quality and hinder the ability to distinguish the object in the quantum illumination AND-image with accidentals subtracted relative to the quantum illumination AND-image. As a result the quantum illumination imaging scheme that we present here is adapted well for adverse imaging conditions of a strong background and high losses, however, as the level of adversity increases so does the number of frames required. These results strengthen the case for quantum illumination protocol implementation in a variety of fields in which the effects of noise and spoofing of an image or scene may prove troublesome. This result allows the quantum signal to be extracted from the detected signal over a large range of background noise levels relative to the quantum signal used to probe the real scene. Therefore, this work offers routes for the implementation of the quantum illumination protocol in real-world environments for the purposes of realising covert imaging schemes, quantum lidar, and quantum radar. Such applications will become more viable as spatially resolved SPAD arrays^33^ continue to improve with regards to speed and time resolution which will allow accurate time-of-flight measurements to be performed in a spatially resolved manner.

### Data availability

Additional data and materials will be made available online.
